# Insights into the Role of Human Gut Microbiota in *Clostridioides difficile* Infection

**DOI:** 10.3390/microorganisms8020200

**Published:** 2020-01-31

**Authors:** Melina Kachrimanidou, Eleftherios Tsintarakis

**Affiliations:** First Department of Microbiology, Aristotle University of Thessaloniki, Medical School, 54124 Thessaloniki, Greece; ltsintarakis@windowslive.com

**Keywords:** *Clostridioides difficile*, dysbiosis, fecal microbiota transplantation, gut microbiota

## Abstract

*Clostridioides difficile* infection (CDI) has emerged as a major health problem worldwide. A major risk factor for disease development is prior antibiotic use, which disrupts the normal gut microbiota by altering its composition and the gut’s metabolic functions, leading to the loss of colonization resistance and subsequent CDI. Data from human studies have shown that the presence of *C. difficile*, either as a colonizer or as a pathogen, is associated with a decreased level of gut microbiota diversity. The investigation of the gut’s microbial communities, in both healthy subjects and patients with CDI, elucidate the role of microbiota and improve the current biotherapeutics for patients with CDI. Fecal microbiota transplantation has a major role in managing CDI, aiming at re-establishing colonization resistance in the host gastrointestinal tract by replenishing the gut microbiota. New techniques, such as post-genomics, proteomics and metabolomics analyses, can possibly determine in the future the way in which *C. difficile* eradicates colonization resistance, paving the way for the development of new, more successful treatments and prevention. The aim of the present review is to present recent data concerning the human gut microbiota with a focus on its important role in health and disease.

## 1. Introduction

*Clostridioides difficile*(*C. difficile*) is the leading cause of healthcare-associated infections and an important healthcare pathogen; however, *Clostridioides difficile* infection (CDI) epidemiology has changed over the past 20 years, with the emergence of a hypervirulent clone NAP1/027/BI implicated in large outbreaks worldwide in the early 2000s [1,2]. Since the application of fecal transplantation (FMT) suggests a causal relationship between gut microbiota composition and the cure for several diseases, we have come to appreciate the central role of gut microbiota in the development of CDI [3].

The microbiota of the gastrointestinal tract is estimated to consist of 100 trillion of microorganisms (10^14^ microbes), the vast majority of which are found in the large intestine, where their population approaches 10^11^–10^12^ cells/ml, the largest number ever recorded for any natural microbial ecosystem [4,5]. The diverse gastrointestinal microbiota is predominantly composed of bacteria from two major phyla, *Firmicutes* and *Bacteroidetes* [6]. This diverse and complex microbiome serves as a functional expansion of host genomes and is estimated to harbor 50- to 100-fold more genes compared to the host [7].

The human gut microbiota is involved in many functions of the host, such as food processing, adjustment of the gut epithelium development, the synthesis of essential vitamins, and pathogen protection [6,8,9,10]. The role of gut microbiota and their unique metabolites is crucial in conferring the host defense against invading pathogens, colonization, and the regulation of important host functions, including metabolism, the development of immunity and the nervous system.

Although human microbiome research is still at a preliminary stage, findings are promising in terms of clarifying the microbiome–host relationships, their crucial role in disease pathogenesis, as well as their therapeutic value. 

Advances in sequencing technologies, as well as the metagenomics of the human intestinal tract, focus research on the gut microbiome and their correlation with health and disease. Several analyses such as 16S ribosomal RNA (rRNA) sequencing to taxonomically classify the microbial populations and whole-genome shotgun (WGS) metagenomic sequencing of body-site specific whole community DNA provide precious data of human microbiota [11].

A gut microbial imbalance (dysbiosis) may lead to dysfunction of the host, contributing to pathogenesis or progression toward a broad spectrum of diseases such as *C*. *difficile* infection. Undoubtedly, the development of new sequencing techniques and bioinformatic techniques allow the study of the human microbiota and increase the interest in the disturbances or alteration of gut microbiota composition, which can favor *C. difficile* colonization, infection and recurrence.

The purpose of this review is to explore current data and discuss recent investigations specifically related to the role of gut microbiota in the pathogenesis of CDI, focusing on the role of the risk factors for CDI, such as age, antibiotics and proton-pump inhibitors (PPI). Novel therapies derived from microbiome studies, such as fecal microbiota transplantation (FMT) to target CDI, have been reviewed to introduce the hypothesis of symptoms resolution through dysbiosis correction, thus revealing a new scientific approach toward disease treatment.

## 2. *C. difficile* Microbiology and Epidemiology

*C. difficile* is a Gram-positive, obligate anaerobic, spore-forming bacterium. The microorganism exists either in its vegetative form, which is sensitive to oxygen, or in its spore-forming form that survives in difficult circumstances for long periods. *C. difficile* was first isolated in 1935, but it was not until 1978 that it was identified as the causative agent of antibiotic-associated diarrhea and colitis [12]. *C. difficile* causes a broad spectrum of clinical symptoms ranging from mild diarrhea to severe life-threatening colonic perforation and toxic megacolon [13,14]. 

Nowadays, *C. difficile* has become a leading cause of hospital-acquired infections and CDI represents a major health problem. The epidemiology of CDI has changed dramatically over the last 20 years. Since 2000, there has been a significant increase in the incidence and severity of CDI in the US, Canada, and Europe, primarily due to the hypervirulent clone PCR ribotype 027/NAP1/BI [2,15]. Mortality rates have also increased worldwide, with an estimated 14,000 deaths annually in the US [16].

Changing epidemiology of CDI has been described not only in hospitals but also in the community. Community-acquired CDI accounts for one-quarter of all diagnosed CDI patients, and usually, these subjects do not have the classic risk profile of patients who develop the infection in a healthcare facility [17].

However, the majority of hospitalized patients infected by *C. difficile* are asymptomatic carriers, who serve as silent reservoirs for continued *C. difficile* contamination of the hospital environment. *C. difficile* is commonly present in the stools of approximately 3–5% of healthy adults and about 30–70% of infants [18]. 

## 3. *C. difficile* Infection (CDI) Pathogenesis

**Figure 1:**
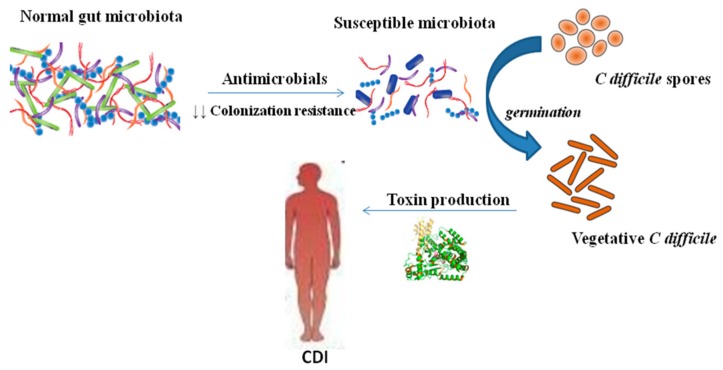
Gut microbiota and *Clostridioides difficile* infection (CDI). Gut microbiota has central role to *C. difficile* infection pathogenesis. ↓ decreased.

*C. difficile* is spread via the fecal–oral route. The organism is ingested either as the vegetative form or as spores, which can survive for long periods in the environment and can penetrate the acidic barrier of the stomach [19]. In the small intestine, spores germinate into a vegetative form [20]. The gut microbiota act as a protective barrier against the colonization of the intestine with *C. difficile.* This barrier is disrupted when the normal gut microbiota have been altered by antibiotic therapy (Figure 1). After contamination by *C. difficile* spores, spores germinate and vegetative forms multiply, which leads to colonization with *C. difficile*. After colonization, the organism produces and releases the main virulence factors, the two large clostridial toxins A (TcdA) and B (TcdB). TcdA and TcdB are exotoxins that bind to human intestinal epithelial cells and are responsible for inflammation, fluid and mucous secretion, as well as damage to the intestinal mucosa [21]. TcdA is an enterotoxin that is responsible for the activation of inflammatory mediators such as IL-6 and IL-8 of the human intestinal epithelial cells and IL-1, IL-6, IL-8, TNF-α of the monocytes. However, TcdB is a cytotoxin that appears to be required for the pathogenic effect of the microorganism [22]. Some *C. difficile* strains produce an additional toxin, a binary toxin (ADP-ribosyl-transferase, CDT) that consists of two components—CDTa, the enzymatic ADP-ribosyltransferase which modifies actin, and CDTb, which binds to host cells and translocates CDTa into the cytosol [23,24,25]. Basic predisposing factors for the *C. difficile* infection are the intake of antibiotics that disrupt the gut microbiota, an extended hospital stay, and advanced patient age.

## 4. Gut Microbiota: Structure and Function

Gut microbiota consists of members of bacteria, phages, archaea, fungi, viruses, and eukaryotic microorganisms. The microbial colonization of the human gut evolves at birth and is influenced by several factors, including host genetics, modality of birth, age, diet, weight, environment, antibiotic exposure, and hospitalization, among others. However, exposure to antimicrobial agents is considered the most disruptive among these factors [26].

The gut microbiome comprises the totality of microorganisms, bacteria, viruses, protozoa, and fungi, and their collective genomes inhabiting our gut [27,28]. Disruptions in the gut microbiome have been associated with a wide range of important diseases including inflammatory bowel disease, diabetes, obesity, neurodevelopmental disorders, and Crohn’s disease [29,30,31]. The diversity and the abundance of human microbiota, and specifically the gut microbiota, pose a challenge to the researchers that investigate the alterations in its composition over time. The development of new modern qualitative and quantitative techniques based on DNA hybridization and PCR methods allowed the detailed description of the composition of microbiota, and specifically the one of the gut [32]. 

The 16S rRNA gene appears in all bacteria and has both fixed and variable areas (V_1_–V_9_). Bacterial 16S rRNA genes generally contain nine “hypervariable regions” that demonstrate considerable sequence diversity among different bacterial species. 16S sequencing is a rapid and accurate identification method for bacterial and archaeal isolates; however, it is not applicable for several genera and provides relatively good resolution until the genus level. This genomic analysis is significant for the analysis of the microbes directly from their environment, providing information for the nucleotide sequence of genes out of the total microbes’ genome [33]. Modern microbiome studies often rely on the analysis of 16S ribosomal RNA sequences for the taxonomic identification of bacterial strains and various microbial communities.

The dominant gut microbial phyla are *Firmicutes, Bacteroidetes, *Actinobacteria*, *Proteobacteria*, *Fusobacteria*, and *Verrucomicrobia**, with the two phyla *Firmicutes and Bacteroidetes* representing 90% of gut microbiota [34]. In the human large intestine, *Bacteroides* are abundant and the *Lachnospiraceae* and *Ruminococcaceae* bacteria families that belong to the phylum *Firmicutes* are quite abundant, typically representing 50%-70% of the bacteria, based on the analyses of 16SrRNA [35]. In the small intestine, the prevailing phylum are *Firmicutes* and *Actinobacteria* [36]. The composition of gut microbiota is characterized by strong differentiation in each person. Studies have shown that it may be categorized in three gut types, depending on the prevalence of the genus: *Bacteriodes* (gut type 1), *Prevotella* (gut type 2), or *Ruminococcus* (gut type 3) [37,38]. A study of consecutive stool samples from 207 individuals showed that despite the alterations in the overall composition of stool at various time points, the participants maintained specific strains, verified by identical mutations (SNP) in these microbes at various time points [38]. Therefore, in each person’s microbiota, there are bacterial strains that characterize their microbiome, and which cannot be easily replaced [39].

The gut microbiota provides many benefits to the host. They confer colonization resistance against pathogens, develop the host immune response, and exert important metabolic functions [40,41]. Recent studies have started to illuminate the mechanisms by which different bacterial species confer resistance against infections. These mechanisms vary from direct inhibitory molecule production and nutrition competition to indirect routes through the stimulation of local innate lymphoid cells (ILCs), myeloid cells, or T- and B-cell responses. ILCs have been implicated in protection against different intestinal pathogens, and specifically, ILC1s protect against *C. difficile* through interferon (IFN)-γ production [42].

*Bacteroidetes* have the ability to break down host glycans and non-digestible carbohydrates. *Firmicutes,* especially members of the *Clostridium* genus, have demonstrated their ability to degrade polysaccharides and ferment amino acids [35]. The availability of carbohydrates, supplied by host and diet, in the gastrointestinal (GI) tract, plays a major role in shaping the structure–function of the microbiota. Some gut bacteria have the ability to forage on mucin glycans within the GI tract. The *O*-glycan structures present in mucin are diverse and complex and the ability to metabolize these mucin *O*-linked oligosaccharides is probably the critical factor in determining which bacterial species colonize the mucosal surface [43]. Mucin-degrading bacteria are privileged to influence the host immune response [44]. Recent findings showed that alterations in mucosal carbohydrate availability affect the composition of microbial species [45,46]. Anaerobic microorganisms in the gut play a dominant role in fermenting complex carbohydrates and amino acids into short-chain fatty acids (SCFAs). SCFAs, specifically butyrate, are important for regulating the gene expression, inflammation, differentiation, and apoptosis of the host epithelial cells [47,48]. Moreover, members of the gut microbiota are important for bile acid metabolism. Bile acids are synthesized from cholesterol by hepatic enzymes, are important for lipid digestion, and also modulate lipoprotein, glucose, drug and energy metabolism [47,48]. While most BAs are efficiently absorbed and recycled back to the liver, 5% of total BA serve as a substrate for bacterial metabolism in the GIT and constitute the major route for cholesterol excretion [49]. Bacterial deconjugation of bile salts makes them less soluble and less efficiently reabsorbed, resulting in higher excretion of free BA into the feces [50]. Although several hypotheses have been proposed, the benefits of this transformation to the bacterium are still a matter of conflict and appear to vary between bacterial isolates [51].

However, gut dysbiosis, the imbalance in a microbial ecosystem characterized by a shift in the composition or function of microbes, can result in pathogenesis.

## 5. Disruption of the Microbiome and CDI Risk Factors

The alteration in the composition of the normal gut microbiota is affected by both physical factors (age, environment) and external factors (nutrition, use of antibiotics, use of proton pump inhibitors-PPIs), which make it vulnerable to its colonization with pathogenic strains, such as toxigenic strains of *C. difficile*.

### 5.1. Gut Microbiota and Antibiotics

The use of antibiotics remains the primary risk factor for the development of CDI, reducing colonization resistance against *C. difficile* via the bio-conversion of primary bile acid to secondary bile acids in the small intestine, as evidenced in studies in experimental animals, as well as in humans [52,53]. Antibiotics alter the structure of the gut microbiota and reduce their diversity, which then alters microbial metabolism in the intestine. These alterations are traceable during the administration and several days after the discontinuation of an antibiotic [54], depending on the administered antimicrobial as well as on the person’s microbiota [55,56]. The restoration of the gut microbiota appears after the discontinuation of antibiotics, while the recovery of the initial composition can be incomplete. Dethlefsen et al. found that although the restoration started within a few weeks after the discontinuation of the administered antibiotic, the new composition did not include all bacterial genera that had been found prior to the initiation of treatment [55,56]. Various animal models have been developed to improve our understanding of the pathogenicity of *C. difficile.* In 2008, Chen et al. developed a mice model that resembles the human infection with *C. difficile* using five antibiotics (gentamicin, kanamycin, colistin, metronidazole, vancomycin) [56]. Reeves et al. used Chen’s model and found a decrease in *Bacteroidetes* and *Firmicutes*, specifically in unidentified *Lachnospiraceae* and an increase in *Proteobacteria* and specifically in the members of the *Enterobacteriaceae* family as compared to healthy control subjects [57]. The same investigators also examined in mice the role of *Enterobacteriaceae* and *Lachnospiraceae* in the colonization resistance to *C. difficile* [58]. They found that members of the bacteria family *Lachnospiraceae* were able to suppress *C. difficile* colonization, toxin production, and disease in vaccinated mice with *Lachnospiraceae*. In addition, mice treated with cefoperazone exhibited an increase in their gut microbiota, in the *Firmicutes* and *Proteobacteria* and more specifically in the *Lactobacillaceae* and *Pseudomonadaceae* families, respectively [57]. Similarly, Buffie et al. found that treatment with clindamycin only demonstrated as prevalent phylum the *Proteobacteria* (*Enterobacteriaceae* family), with a simultaneous decrease in the genera *Bifidobacterium*, *Clostridium* and *Bacteroides* [59]. The specific alterations in the gut microbiota of the mice have been associated with the loss of colonization resistance to *C. difficile.*

However, all categories of antibiotics have been associated with CDI; the antimicrobials most commonly associated with CDI are fluoroquinolones, cephalosporins, and clindamycin [60]. 

A recent study conducted by Antharam et al. compared the gut microbiota of 40 healthy control subjects, 36 patients with diarrhea associated with antibiotics (antibiotic-associated diarrhea, AAD) and 39 patients with CDI. In the AAD and CDI cases, as compared to the healthy control subjects, a decrease was found in the bacteria that produce butyric acid from the *Ruminococcaceae* and *Lachnospiraceae* families and the *Clostridium* clusters IV and XIVa. Furthermore, in CDI cases, an increase was found in the strains *Enterococcus, Veillonella,* and *Lactobacillus*, and in members from the class *Gammaproteobacteria* [61]. In another comparative study of alterations to the intestinal microbiota by Manges et al., hospitalized patients with CDΙ exhibited an increase in the phyla *Firmicutes, Proteobacteria,* and *Actinobacteria* and a decrease in *Bacteroidetes*. The above patients also exhibited an increase in the *Lactobacillaceae* and *Enterococcaceae* families [62]. Rea et al. compared the gut microbiome of patients with positive cultures for *C. difficile* (carriers) to that of patients with negative cultures for *C. difficile.* The positive patients had a decrease in *Bacteroides, Prevotella,* and *Bifobacteria* genera and an increase in the members of the *Lactobacillaceae* and *Enterobacteriaceae* families [63,64]. 

Antibiotics affect the total number and the proportions of metabolites in the intestine by altering the gut microbiota. Bacterial fermentation is particularly modified, leading to a decrease of short-chain fatty acids and an excess of carbohydrates and amino acids [64,65], *C. difficile* could utilize primary bile acids for stimulating germination process and, therefore, would also be able to colonize and grow [66]. The primary bile acids assist in digesting fat, after being produced in the liver and reabsorbed from the small intestine. A small amount of primary bile acids that is not reabsorbed is passed into the colon, where they are metabolized into secondary bile acids by the normal gut microbiota. These secondary bile acids inhibit *C. difficile* growth [66].

### 5.2. Gut Microbiota and Age

Advanced age (>65 years) is an independent risk factor for the development of CDI. The age-related normal alterations in the gastrointestinal tract, as well as the dietary habits, the more frequent hospital visits, and the human immune response impact the composition of gut microbiota. While the gut microbiota of healthy adults appears to be relatively stable, in individuals over the age of 65, a reduction of the protective species of the *Bifidobacteria* and some members of the *Firmicutes* has been found, as well as an increase in *Bacteroidetes* and the more harmful phylum, such as *Proteobacteria* [63,67]. 

### 5.3. Gut Microbiota and Proton Pump Inhibitors (PPIs- Gastric Acid Suppressants)

Proton pump inhibitors (PPIs) are a class of medications used to treat common gastrointestinal conditions. They work by blocking an enzyme in the wall of the stomach to suppress the gastric acid production [68,69]. These gastric acid suppression agents may decrease the colonization barrier against *C. difficile* by increasing gastric pH [70]. The use of PPIs, particularly in combination with antibiotics, disrupts the gut microbiota and is associated with the development of CDI. In vitro studies have shown that the PPIs can affect the development of *Lactobacillus*, and at the same time cause a decrease in the *Bacteroidetes* and an increase in the *Firmicutes* at the phylum level, and an increase in *Holdomania filiformis* and a decrease in *Pseudoflavonifractor capillosus* at a species level [71]. Persons who develop CDI are known to have a decrease in the diversity of the fecal microbiota compared to healthy controls and are more likely to have a relative increase in *Firmicutes* as well as a reduction in *Bacteroidetes* [72]. The alteration in the ratio of *Firmicutes* to *Bacteroidetes* may pre-dispose to the development of CDI. 

### 5.4. Inflammatory Bowel Disease (IBD) and CDI

There is increasing evidence of the pathogenic implication of host microbiota in inflammatory bowel disease (IBD). 

**Figure 2:**
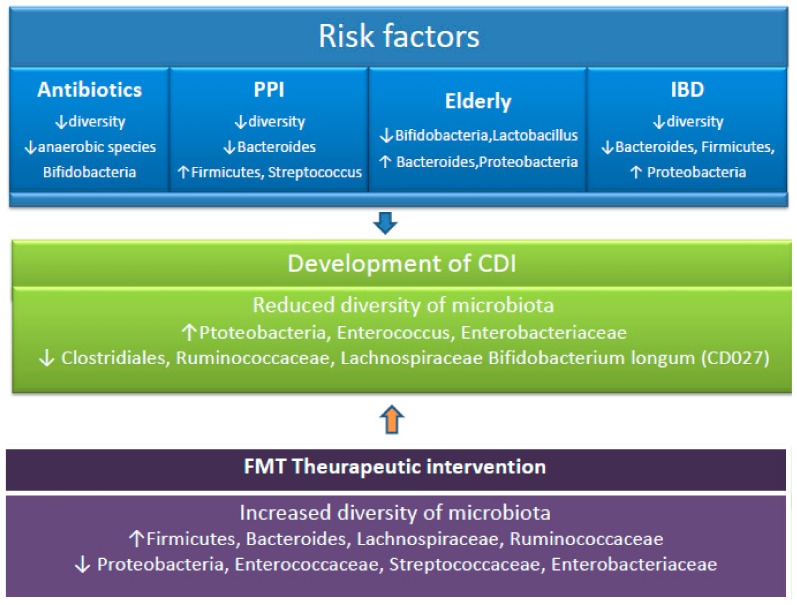
Taxonomic alterations associated with CDI risk factors. Following exposure to CDI risk factors, the microbiome is altered. Disruption of the microbiota due to factors such as antibiotic use, drugs, and prolonged age can lead to the development of CDI. Following fecal microbiota transplantation, the structure and function of the gut microbiota is restored. PPI, proton pump inhibitor. CDI, *Clostridioides difficile* infection. FMT, fecal microbiota transplantation. IBD, inflammatory bowel disease. ↑ increased. ↓ decreased.

Patients who suffer from other gastrointestinal diseases run a possible higher risk of occurrence of *C. difficile* infection [73]. The inflammatory bowel disease (IBD) has been studied in relation to CDI and reduced diversity of the *Firmicutes* and the *Bacteroidetes* in the gut microbiome of these patients was found [74]. In addition, the microbiome of patients with IBD has been associated with the existence of many potentially pathogenic bacteria, mainly *Proteobacteria* [75]. (Figure 2). Clayton et al. evaluated the outpatients with IBD who were in clinical remission and had no recent exposure to antimicrobials, corticosteroids, immunomodulatory drugs, or hospitalizations [76]. These patients were carriers of *C. difficile* by 8.2%, in comparison with 1% of the healthy outpatient population. The immune response of the host also has the ability to regulate the microbiome. Patients with IBD are at increased risk of developing CDI, have worse outcomes of CDI, and also higher rates of recurrence [77]. The inflammatory products, such as antimicrobial peptides lipocalin-2 and calprotectin, deprive the bacteria of iron and zinc essential to their growth, potentially affecting the development of the microorganisms [78].

A relevant study conducted by Andersen et al. reports that IBD activity influences gut microbiota, and also the disease severity and treatment have an effect on the microbial community of the gut. Their results suggested that gut microbiota alteration (dysbiosis) in IBD patients is not only related to current activity but also to the course of the disease [79], providing a favorable environment for the colonization of the gut with *C. difficile* and the subsequent CDI.

## 6. Alterations in Gut Microbiota in CDI

The normal gut microbiota plays a key role in the prevention of infection by increasing colonization resistance against *C. difficile.* The intestinal colonization resistance against pathogens is achieved either with immediate inhibition of the pathogens by substances produced by the microbiota (bacteriocins, antibiotics, short-chain fatty acids (SCFA)) or with competition for the available nutrients or indirectly through the stimulation of the immune defense mechanisms [80,81]. The decrease in the colonization resistance leads to the eradication of certain species or genera, a decrease in the diversity of the microbiota, and alterations in the availability of nutrients and the physiology of the mucus [81]. The exact mechanisms with which the gut microbiota limits the development of *C. difficile* are associated with the alteration of the metabolism of the bile salts, the competition for nutrients, and the decrease in bacteria that produce butyric acid [50,82]. The impact of gut microbiota in the intestinal metabolism (including carbohydrate fermentation, regulation of amino acid metabolism, protein digestion, and lipid metabolism) is important, converting luminal compounds into secondary metabolites, which can be either beneficial or harmful to the host.

Alterations in gut microbial composition have been described for CDI patients, including lower species richness and microbial diversity than in healthy individuals [83,84], with the dominance of *Proteobacteria* and specifically an abundance of *Enterobacteriaceae* [85]. 

However, alterations in gut microbial composition in patients with asymptomatic *C. difficile* colonization have not been clearly described. The intestinal microbial communities of patients with CDI differ from *C. difficile* carriers, and therefore it is suggested that the absence, presence, or abundance of specific bacterial taxa is a possible cause for the development of CDI or *C. difficile* colonization [86,87]. *C. difficile* carriers were found to have fewer *Proteobacteria* and a larger proportion of *Firmicutes* and *Bacteroidetes* than CDI patients, so as to closely resemble healthy individuals [84]. The role of asymptomatic *C. difficile* colonization in the development of CDI is still a controversial matter of debate. 

Horvat et al. demonstrated that *C. difficile* could have a strain-dependent impact on gut microbiota [88]. The study suggested that the association of *C. difficile* colonization and/or infection with a decrease in gut microbiota diversity is not only a predisposing condition [89] but also a consequence of *C. difficile* infection [88]. 

According to Khana et al., the gut microbiome predicts the treatment response and recurrence potentially in primary CDI. Patients who responded to treatment had an increase in *Ruminococcaceae, Rikenellaceae, Clostridiaceae, Bacteroides, Faecalibacterium,* and *Rothia* compared to nonresponders. Moreover, patients with recurrent CDI had statistically significant increases in *Veillonella, Enterobacteriaceae, Streptococci, Parabacteroides,* and *Lachnospiraceae* compared to patients without recurrence [90].

### 6.1. Bile Acids and C. difficile

Gut microbiota also plays an important role in the co-metabolism of bile acids with the host. The primary bile acids that are synthesized in the liver consist mainly of cholic and chenodeoxycholic acid, which are conjugated with taurine or glycine [91,92]. The fraction of the bile acids that is not reabsorbed in the ileum (<5%) passes through the colon, where it undergoes bacterial metabolism; the primary bile acids are metabolized in secondary ones, such as lithocholic and deoxycholic respectively [49,50,93]. In vitro studies have shown that the conjugated and deconjugated forms of cholic acid cause the spore germination of *C. difficile,* while chenodeoxycholic acid inhibits it [94,95]. Lithocholic acid inhibits the germination of the spores, whereas deoxycholic acid fosters it, but it is toxic for the vegetative form of *C. difficile* [94,96]. When the gut microbiota is disrupted by the intake of antibiotics, the primary bile acids are not transformed into secondary ones [97], with an increase in the proportion of the cholic to chenodeoxycholic derivatives [97] due to faster absorption of the latter by the epithelium of the large intestine. This entire procedure causes the germination of the spores and the development of *C. difficile* [20]. Primary bile acids (cholate derivatives), which are in high concentrations in the small intestine, serve as germinant for *C. difficile* spores, whereas secondary bile acids (deoxycholate) in low concentration in the hind gut inhibit vegetative growth of *C. difficile*. Antibiotic administration reduces the gut microbial communities’ diversity, leading to a significant reduction in microbial bioconversion of primary bile acids into antimicrobial secondary bile acids, leading to reduced inhibition of *C. difficile* vegetative growth, allowing *C. difficile* overgrowth and colonization [98]. As a result, there is a higher susceptibility of the host toward CDI.

### 6.2. Availability of Substrate Sources and C. difficile

Antibiotics can affect the availability of nutrients in the intestine in various ways. First, by decreasing the diversity of the gut microbiota, they reduce the competition for limited resources, fostering the development of micro-organisms. At the same time, the lysis of the sensitive to antibiotics bacteria releases carbon sources that are consumed by other members of the microbial community [99]. Wilson and Perini found that the components of the mucin (sialic acids, Ν-acetylglucosamine, and Ν-acetylneuramanic acid) which are abundant in the intestine, positively affect the in vitro development of *C. difficile* [100]. Under normal conditions, the gut microbiota microorganisms compete for the consumption of sialic acids that are released by the intestinal mucosa. However, when the microbiota is suppressed by antimicrobials, the sialic acid catabolism by *C. difficile* provides an advantage in *C. difficile* proliferation [46]. 

### 6.3. Butyric Acid and C. difficile

Butyric acid is a source of energy for the intestinal cells and plays a key role in the regulation of the proliferation, the intestinal epithelial cells’ diversity, and in preserving the integrity of the gut epithelium [101]. It protects the host from infection by increasing the defense barriers through the stimulation of the production of mucin and antimicrobial peptides [102]. SCFA have been proven to inhibit the in vitro development of *C. difficile*, and has an anti-inflammatory effect. The decrease in the bacteria that produce butyric acid can alter the defense of the host against *C. difficile,* and can increase the sensitivity to CDI [61]. The genera *Roseburia* and *Coprococcus* that belong to the *Lahnospiraceae* family and also *Faecalibacterium prausnitzii*, a major member of the *Firmicutes*, are important members of the gut microbiota and are involved in the production of butyric acid [103]. In a relevant study by Antharam et al., several members of the *Ruminicoccaceae* and *Lahnospiraceae* families appeared decreased in an infection from *C. difficile* [61]. Beneficial bacteria, particularly butyrate-producing bacteria (*Lachnospiraceae* and *Ruminococcaceae*), are present in larger proportions in microbiota without *C. difficile*. These bacteria are usually found in healthy subjects and are thought to provide colonization resistance against CDI [80].

## 7. Restoration of the Gut Microbiota as Therapeutic for CDI

Improved understanding of antibiotic-induced microbiome dysbiosis in the pathogenesis of CDI has given rise to the development of new promising therapeutic approaches that involve the restoration of gut microbiota, such as fecal microbiota transplantation (FMT). 

The administration of antibiotics, such as vancomycin, metronidazole, or fidaxomicin, is typically successful in relieving CDI symptoms. However, the treatment of recurrent infections from *C. difficile* is a great therapeutic challenge, where the transplantation of gut microbiota seems particularly promising in restoring gut homeostasis [104]. After CDI treatment, the risk of a recurrence within 8 weeks is 15–25% and is further increased up to 60% in patients with multiple recurrences [105]. Fecal microbiota transplantation (FMT) was used as a treatment, aiming at re-establishing colonization resistance in the host’s gastrointestinal tract by replenishing the gut microbiota. Restoration of the composition of the microbiota and community structure leads to restoration of its function, including colonization resistance, gut homeostasis, and physical barrier defense [106]. 

FMT is becoming a mainstream treatment for recurrent CDI. Microbiota replacement therapies for CDI include capsule-based and enema-based therapies [107]. 

In enema based therapy, feces from a healthy donor are inserted into the colon of a patient with recurrent disease after filtering through a nasogastric oral tube, rectal enema, or colonoscopy [108,109]. The ideal recipients of FMT are patients over the age of 18 with concurrent CDI and failure of appropriate antibiotic treatment. The number of concurrent infections required to conduct fecal transplantations has not been clearly defined. However, recent evidence includes cases of patients with three or more incidents of CDI despite appropriate treatment, or two or more incidents of CDI with hospitalization and significant morbidity, patients with moderate CDI irresponsive to treatment with oral vancomycin for over a week, or patients with severe CDI irresponsive to oral vancomycin for 48 hours [110,111,112]. In a randomized study, 93% of the patients that received oral vancomycin and bowel irrigation followed by FMT exhibited a significant decrease in their diarrheal episodes, whereas only 31% of the patients that received only vancomycin and 23% of those who received vancomycin and bowel irrigation had positive outcomes, demonstrating that FMT was significantly more effective for the treatment of recurrent *C. difficile* infection than the use of vancomycin [113]. FMT is an approved treatment for recurrent CDI but there is uncertainty about its use and could represent a treatment option with perspective. The FMT cost is potentially lower in relation to the cost of repeated courses of antibiotics and hospitalization. The identification of symbiotic bacteria of the gastrointestinal tract, which are able to induce protection against *C. difficile*, could lead, in the future, to the use of beneficial bacteria of either one genus or one mixture of symbiotic bacteria to fight the said pathogen and prevent the infection [114]. Following FMT, there is a shift in gut microbiome composition with an abundance of *Bacteroidetes* and a significant reduction of *Proteobacteria*. Van Nood et al. observed an increase in gut *Bacteroidetes* and *Clostridium* cluster IV and XIVa (*Firmicutes*), and a reduction in *Proteobacteria* post-FMT, suggesting the importance of *Bacteroidetes* and the non-pathogenic Clostridia member in suppressing *C. difficile* outgrowth [7]. Konturek et al. also demonstrated a high curing rate (94%) of CDI after FMT application, presenting an increase in beneficial bacteria including *Lactobacillaceae, Ruminococcaceae, Desulfovibrionaceae, Sutturellaceae,* and *Porhyromonadeacae*, along with decrease in aggresive bacteria such as *Enterobacteriaceae* and *Veillonellaceae* [115].

Khanna et al. detected that SER-109 potentially prevents CDI recurrence within an 8-week follow-up period in patients experiencing recurrence, presumably through a change in the gut microbiota and recovery of natural colonization resistance [116]. SER-109 comprises *Firmicutes* spores derived from healthy donor stool. Results from the phase II trial indicate that SER-109 did not have the primary outcome for reducing CDI recurrence overall. However, in high-risk populations (those 65 years or older), SER-109 treatment showed reduced recurrence rates (45% versus 80% recurrence risk) [117]. There is also another oral microbiome therapeutic called SER-262, in contrast to SER-109, which is a defined microbial community consisting of the spores of anaerobic, commensal bacteria produced by in vitro fermentation and is not an undefined consortium derived from human stool [118]. SER-262 is currently being tested in a phase I trial for adult patients to prevent recurrent CDI. Furthermore, the understanding of metabolic pathways used by symbiotic microbes in order to compete with pathogenic microbes can lead to the genetic modification of the symbiotic microbes with an increased ability to limit the colonization with pathogens. Such a development would enhance our therapeutic arsenal to fight intestinal infections, such as these are caused by *C. difficile*. Moreover, it has been estimated that the change in the diet and the use of prebiotics or probiotics could possibly restore the colonization resistance against *C. difficile* [119,120]. Reducing antimicrobial use would arguably reduce antimicrobial-resistant organisms. Clearly, hand decontamination through proper handwashing and glove utilization may impact on controlling *C. difficile* transmission within healthcare environments.

A comprehensive understanding of the gut microbiota composition, in both states of health and various diseases, is needed for the development of future approaches for microbiota modulation and for developing targeted therapies.

## 8. Conclusions

*C. difficile* is a significant cause of morbidity and mortality worldwide. Although CDI pathogenesis is relatively well known, it is important to better understand the role of the gut microbiota in the prevention and outcome of disease and also the value of FMT in CDI. The role of gut microbiota is prevalent throughout the entire life-cycle of *C. difficile*, from spore transmission and germination into infection development. Several studies have described the composition of the gut microbiota in subjects that develop colonization or infection from *C. difficile*. While the exact reasons for the loss of colonization resistance remain unclear, comprehensive bacterial replacement during FMT has shown that restoration of certain components of the microbiota is effective in treating recurrence. Decreased microbial diversity is associated with recurrent disease. Future research is needed to find the specific community members needed to restore colonization resistance. However, the interactions among host, pathogen, and microbiota in CDI present multiple opportunities for the development of novel therapies that target specific steps in pathogenesis. As such, emerging therapies can reduce the risk of CDI by lowering the effect of systemic antibiotics, decreasing the levels of primary bile acids, increasing secondary bile acids, and restoring the native gut microbiota. 

Insights gained from human gut microbiota have revealed novel therapeutic options for CDI. New techniques, such as post-genomics, proteomics, and metabolomics analyses, can possibly determine the way in which *C. difficile* eradicates the colonization resistance in the intestine, paving the way for the development of new, more successful treatments and prevention.
